# Comparative Chloroplast Genomes of Photosynthetic Orchids: Insights into Evolution of the Orchidaceae and Development of Molecular Markers for Phylogenetic Applications

**DOI:** 10.1371/journal.pone.0099016

**Published:** 2014-06-09

**Authors:** Jing Luo, Bei-Wei Hou, Zhi-Tao Niu, Wei Liu, Qing-Yun Xue, Xiao-Yu Ding

**Affiliations:** College of Life Sciences, Nanjing Normal University, Nanjing, China; Universidad Miguel Hernández de Elche, Spain

## Abstract

The orchid family Orchidaceae is one of the largest angiosperm families, including many species of important economic value. While chloroplast genomes are very informative for systematics and species identification, there is very limited information available on chloroplast genomes in the Orchidaceae. Here, we report the complete chloroplast genomes of the medicinal plant *Dendrobium officinale* and the ornamental orchid *Cypripedium macranthos*, demonstrating their gene content and order and potential RNA editing sites. The chloroplast genomes of the above two species and five known photosynthetic orchids showed similarities in structure as well as gene order and content, but differences in the organization of the inverted repeat/small single-copy junction and *ndh* genes. The organization of the inverted repeat/small single-copy junctions in the chloroplast genomes of these orchids was classified into four types; we propose that inverted repeats flanking the small single-copy region underwent expansion or contraction among Orchidaceae. The AT-rich regions of the *ycf*1 gene in orchids could be linked to the recombination of inverted repeat/small single-copy junctions. Relative species in orchids displayed similar patterns of variation in *ndh* gene contents. Furthermore, fifteen highly divergent protein-coding genes were identified, which are useful for phylogenetic analyses in orchids. To test the efficiency of these genes serving as markers in phylogenetic analyses, coding regions of four genes (*acc*D, *ccs*A, *mat*K, and *ycf*1) were used as a case study to construct phylogenetic trees in the subfamily Epidendroideae. High support was obtained for placement of previously unlocated subtribes Collabiinae and Dendrobiinae in the subfamily Epidendroideae. Our findings expand understanding of the diversity of orchid chloroplast genomes and provide a reference for study of the molecular systematics of this family.

## Introduction

The orchid family Orchidaceae is one of the two largest families of flowering plants, with over 25,000 species [1] and five recognized subfamilies (Apostasioideae, Cypripedioideae, Epidendroideae, Orchidoideae, and Vanilloideae) [2]. A large number of orchids have significant economic value [3]. For example, some cultivars have been used as cut flowers or potted plants, while others can be utilized as food or medicine because of their nutritious or medical efficacy. Overexploitation and habitat destruction have threatened the survival of many wild orchid species. At the same time, numerous cultivated varieties and crossbreeds have been developed worldwide. Therefore, molecular information on orchids is of interest not only for the study of systematics, but also for species conservation and flower cultivation.

Epidendroideae is the largest of the five orchid subfamilies and includes approximately 20,000 species [1], [2]. Several perspectives on its classification have long been debated [2], [4]–[16]. Burns-Balogh and Funk (1986) have reviewed previous morphological classification systems of Orchidaceae. Freudenstein and Rasmussen (1999) pointed out that most of the previously established classifications have a highly developed tribal and subtribal classification within the Epidendroideae; they first performed a cladistic analysis of Orchidaceae. However, major groups of genera are equivalent to subfamilial groups, and the detailed classifications at the tribal level are not well supported by morphological and anatomical features in most cases [12], [16]. In recent years, molecular data has been used in phylogenetic studies, but some relationships among subtribes or tribes remain questionable. Major disputes were focused on whether some tribes or subtribes were monophyletic, polyphyletic, or paraphyletic; which tribe or subtribe was the most basal; and the locations of Agrostophyllinae, Collabiinae and Dendrobiinae. Limited sampling with few variable loci in most of the common DNA regions has impeded reasonable and robust estimates of phylogenetic patterns. Recent comparative chloroplast (cp) genomics has provided large quantities of data that are useful for selecting pertinent markers to resolve obscure phylogenetic relationships in seed plants [17]–[21]. However, cp genome information is still limited for the Epidendroideae.

In most land plants, the cp genome is a single circular molecule of 120–220 kb that consists of one large single-copy (LSC) region, one small single-copy (SSC) region, and a pair of inverted repeats (IRs). Although gene organization and content are conserved in cp genomes of higher plants, their genome sizes are diverse and depend largely on the extent of gene duplication, small repeats, and the size of intergenic spacers [22]. The information on sequence insertion or deletion, transition or transversion, and nucleotide repeats may help to clarify evolutionary relationships [23]–[27]. To date, cp genomes from seven orchid genera (*Corallorhiza*, *Cymbidium*, *Erycina*, *Neottia*, *Oncidium*, *Phalaenopsis*, and *Rhizanthella*) have been sequenced. The former six genera belong to the subfamily Epidendroideae, whereas the last one falls into the subfamily Orchidoideae. All species in these seven genera are photosynthetic orchids except *Rhizanthella gardneri*, *Corallorhiza striata*, and *Neottia nidus-avis* being nonphotosynthetic plants [17], [28]–[34]. However, the cp genome of the Dendrobiinae, the largest and most economically important subtribe in the Epidendroideae, has not yet been sequenced.

*Dendrobium officinale* Kimura et Migo, a perennial epiphytic herb of the Dendrobiinae, is endemic in moderately damp mountains in China [35]. The stems of *D. officinale* have been widely used as a traditional Chinese medicine (TCM) called “Tiepi Fendou.” The efficacious compounds in *D. officinale* include phenols, alkaloids, coumarins, and polysaccharides [36]; and its medical benefits include stimulation of saliva, improvement in eyesight, warming of the stomach, enhancement of immunity, and inhibition of tumor growth [36]. As a result of its habitat shrinking and human overexploitation, natural populations of *D. officinale* have been progressively destroyed, and in 1992 it was classified as an endangered species in the Chinese Plant Red Book [37]. *D. officinale* has recently been rescued by tissue culture in southern China.

The subfamily Cypripedioideae comprises approximately 155 species in five genera [2]. All Cypripedioideae species have special flowers with a saccate lip, two fertile stamens, a shield-like staminode, and a synsepal composed of fused lateral sepals [38]. Because of its attractive morphological characteristics, this subfamily has been investigated widely in theoretical and applied research. Nonetheless, molecular information on this subfamily is still limited. *Cypripedium macranthos* Sw. is a terrestrial herbaceous plant in the subfamily and naturally distributed in East Asia [39]. Because of the commercial value of its pretty red or pink flowers, it has been cultivated as a potted and garden plant.

In this study, we sequenced the complete cp genomes of *D. officinale* and *C. macranthos* using a next-generation sequencing (NGS) approach. Our objectives were to deepen understanding of the structural diversity of orchid cp genomes and to provide information for resolving uncertain relationships within the Epidendroideae. The cp genomes of seven photosynthetic orchid species (*C. macranthos*, *Cymbidium mannii*, *D. officinale, Erycina pusilla*, *Oncidium* Gower Ramsey, *Phalaenopsis aphrodite*, and *Phalaenopsis equestris*) were compared to elucidate the diversity of gene order, gene content, and genome structure among them. Four regions were filtered according to the sequence divergence of protein-coding genes, and 56 taxa from 36 genera were used as a case study to determine phylogenetic relationships within the Epidendroideae.

## Materials and Methods

### Chloroplast DNA extraction and genome sequencing, assembly, and PCR-based validation

This study was approved by the Ethics Committee of Forestry Bureau of Zhejiang Province and Nanjing Normal University, China. We collected seeds of *D. officinale* from Yandang experimental base of Zhejiang Branch, College of Life Sciences, Nanjing Normal University.

Young leaves of *D. officinale* were taken from 6-month-old seedlings grown in a greenhouse. Intact chloroplasts were isolated using the Percoll gradient method (22–45%) [40]. Purified chloroplast DNA was extracted according to the 2× CTAB protocol [41]. Fresh leaves of *C. macranthos* were collected from Yunnan Province, China. Total DNA was extracted using a Qiagen DNeasy plant mini kit (Qiagen, Germany). DNA concentration and quality were determined using a NanoDrop 8000 Spectrophotometer (Thermo Scientific, Wilmington, DE). High quality DNA (concentration >300 ng/µl, A260/280 ratio = 1.8–2.0 and A260/230 ratio>1.7) was used for sequencing.

Purified DNA was fragmented and used to construct short-insert libraries (insert size∼500 bp) according to the manufacturer's instructions (Illumina). The short fragments were sequenced using an Illumina Hiseq 2000 sequencing system [42].

The raw reads for *D. officinale* were trimmed with error probability <0.001 and assembled using SOAPdenovo version 1.05 with default parameters [43]. The de Bruijn graph approach was applied to assembly with an optimal *K*-mer size of 79. The contigs shorter than 200 bp were removed. Then the paired-end information was used to join the contigs into scaffolds with the cp genome of *P. aphrodite* (Accession Number: AY916449) as a reference. Gaps among scaffolds were filled using paired-end extracted reads.

The short reads for *C. macranthos* were trimmed with error probability <0.05 and assembled using CLC Genomic Workbench 6.0.1 (CLC Bio, Aarhus, Denmark). The contigs shorter than 200 bp were discarded; others were compared with plant cp genomes in the National Center for Biotechnology Information (NCBI) using BLAST (http://blast.ncbi.nlm.nih.gov) searches. Contigs matching referenced genomes with *E* values <10^−5^ were selected for annotation.

Based on the reference genomes in Orchidaceae [28], [29], gaps and four junction regions between LSC/SSC and IRs were confirmed by PCR amplification and Sanger sequencing using the primers listed in Table S1.

### Genome annotation

Protein-coding and ribosomal RNA genes were annotated using DOGMA (http://dogma.ccbb.utexas.edu/) [44]. The boundaries of each annotated gene were manually determined by comparison with orthologous genes from other orchid cp genomes. Genes of tRNA were predicted using tRNAscan (http://lowelab.ucsc.edu/tRNAscan-SE) [45] and ARAGORN version 1.2 (http://130.235.46.10/ARAGORN/) [46]. The circular genome maps were drawn using GenomeVx, followed by manual modification [47]. The sequencing data and gene annotation were submitted to GenBank with accession numbers KC771275 and KF925434.

### Analyses of RNA editing sites

Thirty protein-coding genes of *D. officinale* and *C. macranth*os cp genomes were used to predict potential RNA editing sites using the online program Predictive RNA Editor for Plants (PREP) suite (http://prep.unl.edu/) [48] with a cutoff value of 0.8.

### Phylogenomic analyses

Sixty-three common protein-coding genes were extracted from 10 cp genomes. Seven photosynthetic orchid species were involved in analyses with *Calamus caryotoides*, *Phoenix dactylifera*, and *Typha latifolia* as outgroups. The GenBank accession numbers of all taxa are shown in Table S2. The *acc*D, *inf*A, *rp*s16, *rps*19, *ycf*1, and *ndh* genes were not included in the data set because they were pseudogenized in some cp genomes. Alignments were performed using the MUSCLE program in Mega 5.03 [49], without including gaps, and start and stop codons. The aligned sequences were concatenated and used for phylogenetic reconstruction.

The ML tree was constructed by means of GTR+G model with raxmlGUI version 1.2 (http://sourceforge.net/projects/raxmlgui/) [50] and a rapid bootstrap value of 1,000. A Bayesian inference (BI) tree was constructed using CAT model with PhyloBayes version 3.2 [51]. Two Independent MCMC chains were run. The first 25% of the cycles were removed as burn-in, and convergence of three chains was checked on the basis of maxdiff <0.3 by following the PhyloBayes manual.

### Sequence divergence of protein-coding genes

To obtain suitable markers for phylogenetic analysis within subfamilies, complete cp genomes of six orchid species (*C. macranthos*, *C. mannii*, *D. officinale, E. pusilla*, *O*. Gower Ramsey, and *P. aphrodite*) were applied. The average pairwise distances of nucleotide and protein substitutions for 68 protein-coding genes were estimated using Kimura's two-parameter model and p-distance, respectively, in Mega 5.03 [49].

### Phylogenetic application of cp genomes, a case study on the Epidendroideae

We selected the Epidendroideae as an example for phylogenetic analysis. Data sets for four incomplete gene sequences (*ycf*1, *mat*K, *ccs*A, and *acc*D) were obtained for 56 taxa from 36 genera. The data matrix included 11 subtribes and one tribe in the Epidendroideae, with *C. caryotoides*, *P. dactylifera*, and *T. latifolia* as outgroups. Six taxa from two additional orchid subfamilies were used as internal checks. Sequences from 10 of the taxa were extracted from complete cp genomes; sequences from the other 46 taxa were obtained by PCR amplification and sequencing of PCR products with an ABI PRISM 3730XL DNA analyzer (Applied Biosystems). Primers for *acc*D and *ccs*A were designed using Primer Premier version 6 [52] based on homologous sequences from orchid cp genomes (Table S3). All newly generated sequences were deposited in GenBank with accession numbers KF361524-KF361707. Sources of species and GenBank accession numbers are indicated in Table S4. These regions were aligned separately using Mega 5.03 [49] with manual modifications, and gaps were coded as “-.” Sequence information was analyzed using Mega 5.03 and DnaSP version 5.0 [53]. The combined matrix was utilized for phylogenetic analyses. Modeltest version 3.7 [54] was employed to select the best nucleotide substitution model under the Akaike Information Criterion (AIC); the GTR+I+G model was chosen as the best fit for our data set. The ML and BI analyses were performed according to the same protocol as that used for phylogenomic analyses.

## Results

### Sequencing and genome assembly

The raw Illumina paired-end sequencing of *D. officinale* produced 350 Mb of data. After quality trim, 210 Mb of data remained with an average read length of 80 bp. The subsequent *de novo* assembly produced 13 scaffolds, 12 of which were >2 Kb and the scaffold N50 size was 84,551 bp. The average coverage depth was 1,400×. These scaffolds were used for the following assembly.

We sequenced 2.5 Gb of Illumina paired-end reads for *C. macranthos* (average read length of 90 bp). The initial assembly included 12,148 contigs. After compared with plant cp genomes, 41 contigs were obtained with *E* values<10^−5^ and mean coverage depth  = 26×. Four of these contigs were larger than 10 kb with average depth coverage 129×, resulting in a nearly complete draft genome. After assembly and gap closure, two complete chloroplast genomes were obtained.

### Characteristics of the chloroplast genomes of *Dendrobium officinale* and *Cypripedium macranthos*

**Figure 1:**
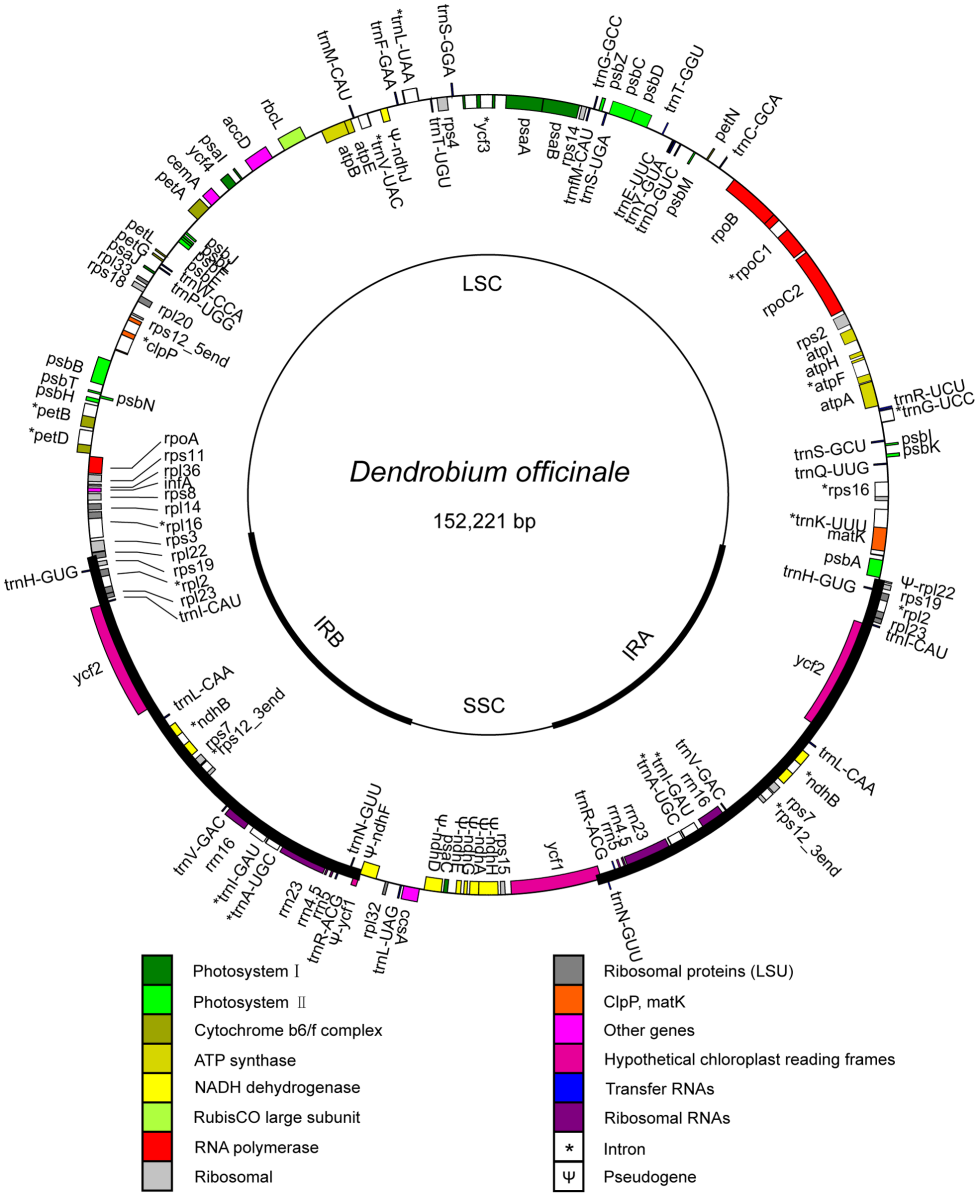
Map of the chloroplast genome of *Dendrobium officinale*. Thick lines indicate inverted repeats (IRs). Genes shown inside the circle are transcribed clockwise, and those outside the circle are transcribed counterclockwise.

**Figure 2:**
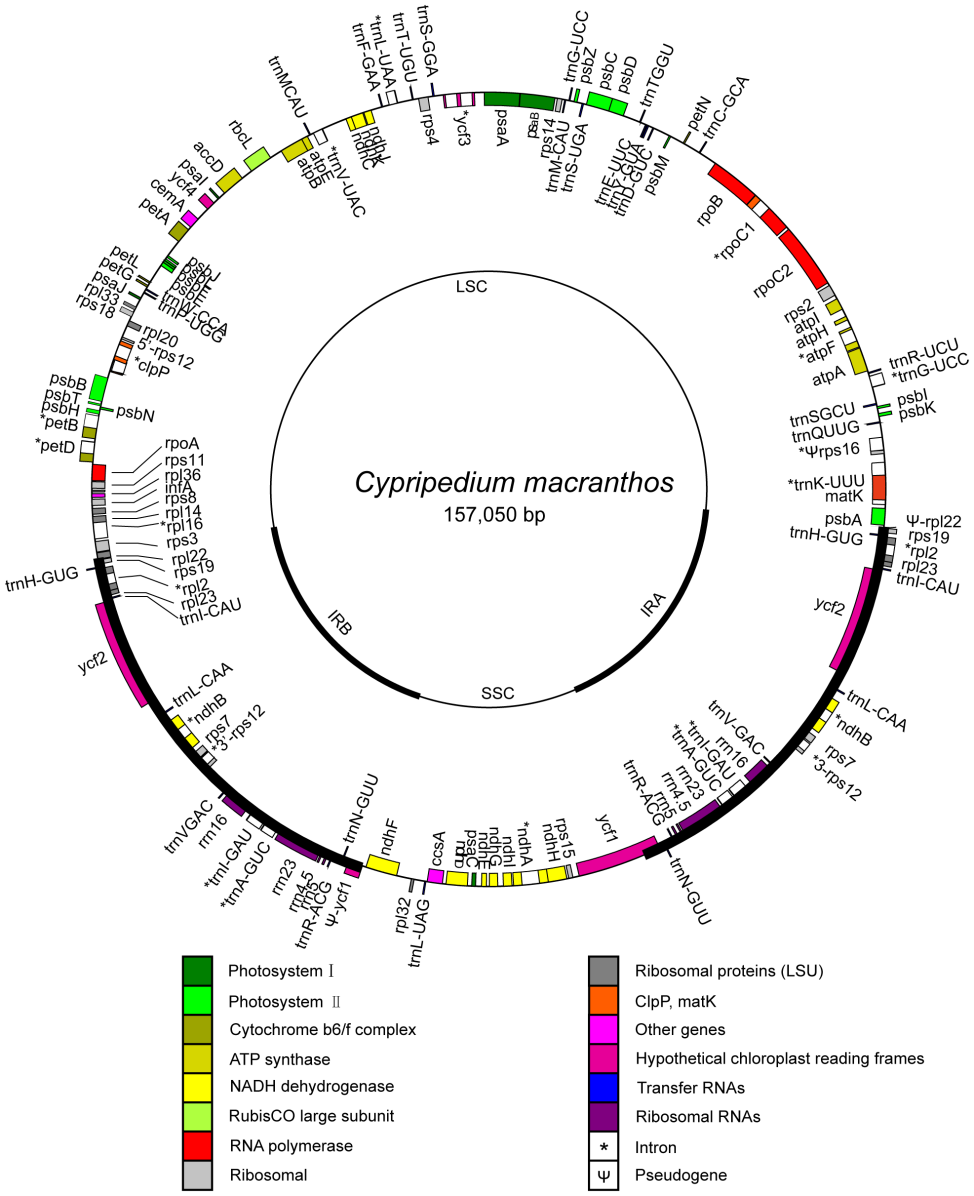
Map of the chloroplast genome of *Cypripedium macranthos*. Thick lines indicate inverted repeats (IRs). Genes shown inside the circle are transcribed clockwise, and those outside the circle are transcribed counterclockwise.

The complete cp genomes of *D. officinale* and *C. macranthos* were circular, having 152,221 and 157,050 bp, respectively. Similar to other angiosperms, both cp genomes were AT-rich (62.53% and 62.17%, respectively). The *D. officinale* plastome contained 110 different genes, of which 91 were single-copy genes and 19 were duplicated genes (Fig. 1). Its cp genome consisted of 76 protein-coding genes, 4 rRNA genes, and 30 tRNA genes. *C. macranthos* encoded 113 different genes (94 single-copy and 19 duplicated genes). The *C. macranthos* cp genome included 79 protein-coding genes, four rRNA genes, and 30 tRNA genes (Fig. 2). The gene content of the *D. officinale* cp genome was relatively conserved compared with other known orchid cp genomes. The gene content of the *C. macranthos* cp genome was also relatively conserved with the exception of the following. A coding sequence (CDS) of *inf*A (coding for translation initiation factor) was interrupted because of a 5-bp deletion (53 bp downstream of the start codon). This gene was lost from *Discorea* in monocots [55]. At the N terminus of *rps*19, a surplus nucleotide A in the poly (A) tract interrupted the open reading frame (ORF), causing a frameshift. Furthermore, we recognized *rps*16 as a pseudogene because a partial intron and the second exon were missing in it.

### Potential RNA editing sites

In the present study, potential RNA editing sites were predicted for 30 genes; as a result, a total of 51 RNA editing sites were identified in genes of *Cypridium* and *Dendrobium* (Table S5). No potential editing sites were identified in seven genes (*pet*D, *pet*G, *pet*L, *psb*B, *psb*E, *psb*L, and *rpl*23) in both cp genomes. Of the 51 editing sites, 9 (17.6%) and 42 (82.4%) were located at the first and the second codon position, respectively, in *Cypripedium*; 8 (15.7%) and 43 (84.3%) were located at the first codon and the second codon position, respectively, in *Dendrobium*; but no editing sites were found at the third codon position. Just as in other terrestrial plants, the editing types in *Cypripedium* and *Dendrobium* were all C-to-U [56]–[58]. The amino acid conversion S to L occurred most frequently, while P to S and R to C occurred least. Thirty-four common RNA editing sites were shared in genes of the two species. We also observed RNA editing (C to U conversion) in the initiation codon of *rpl*2 transcripts of *D. officinale*, which is a common phenomenon among angiosperms and has been verified in *P. aphrodite* and *R. gardneri* [28], [30].

### Phylogenomic analyses of the seven orchids

**Figure 3:**
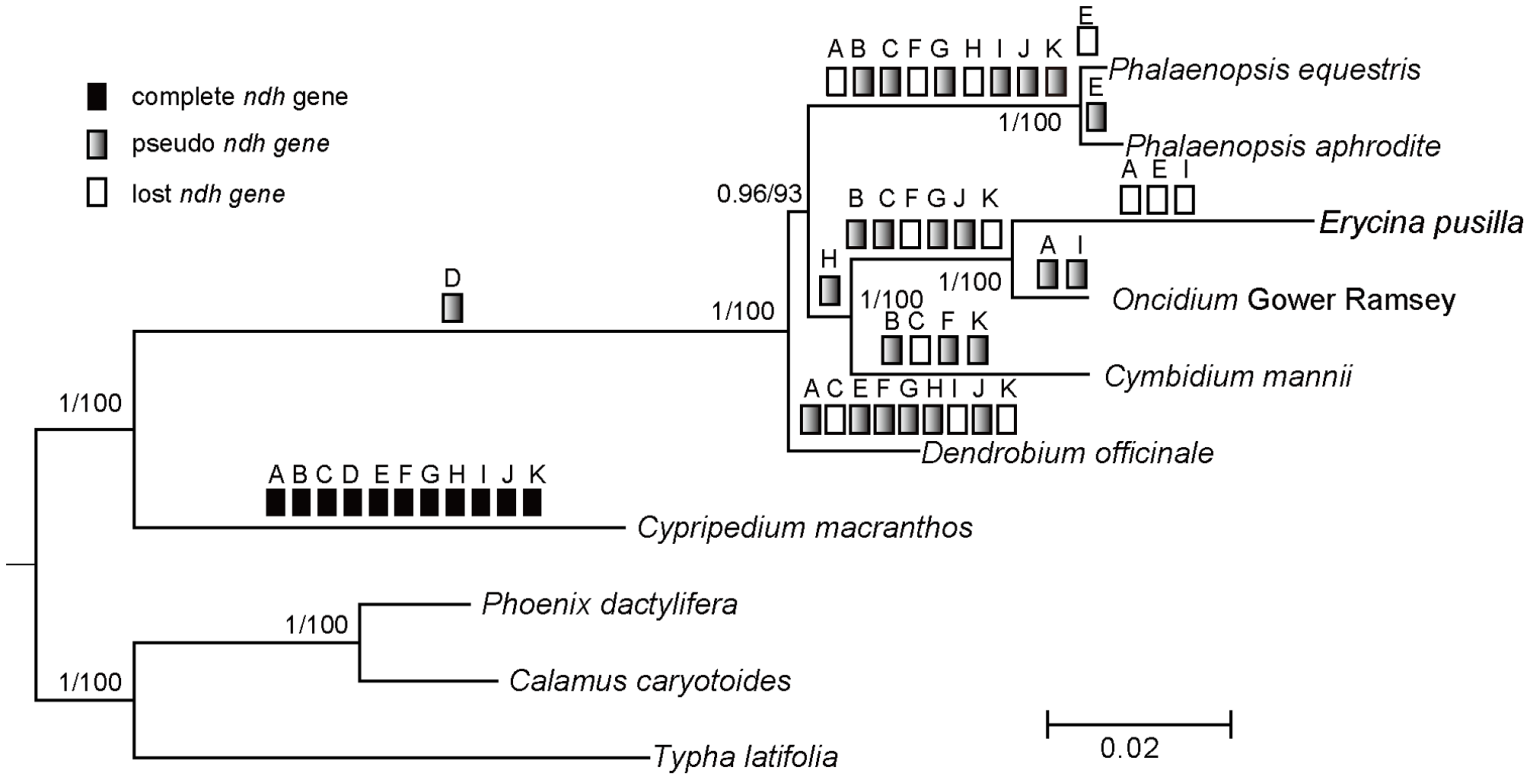
Phylogenomic tree based on 63 protein-coding genes. Only the BI tree is shown because BI and ML trees had identical topologies. Numbers near branches are posterior probabilities for BI analysis and bootstrap values for ML analysis. The degenerate *ndh* genes are mapped in the tree. Solid, empty, and gray bars show the distribution of *ndh* genes in orchids, indicating intact, lost, and pseudogenized genes, respectively.

Our phylogenomic construction was based on 63 protein-coding genes of cp genomes, and the aligned data set comprised 47,736 bp. The BI and ML trees had the same topology (Fig. 3), demonstrating that *Cypripedium* (Cypripedioideae) was sister to the Epidendroideae. In the Epidendroideae, *Dendrobium* was sister to other species, and *Cymbidium* and *Oncidium*-*Erycina* were sister to *Phalaenopsis*.

### Comparison of chloroplast genomes of seven photosynthetic orchids

Six photosynthetic orchid species representing four subtribes of the subfamily Epidendroideae—Cymbidiinae (*C. mannii*), Aeridinae (*P. aphrodite* and *P. equestris*), Oncidiinae (*O.* Grower Ramsey and *E. pusilla*), and Dendrobiinae (*D. officinale*)—were compared with the reference species *C. macranthos* in the organization and gene content of their cp genomes. The seven cp genomes ranged from 146,484 to 157,050 bp (average length = 150,307±4,889 bp) (Table 1). Compared with *C. macranthos*, the other taxa had reduced IR length. The organization of the cp genomes of the Epidendroideae was similar to that of the *C. macranthos*, except for three sequences: *Ψycf*1-*ndh*F, *ndh*C-*ndh*J, and *ndh*D-*ndh*H. Variations in *Ψycf*1-*ndh*F sequence were due to reductions in the lengths of *Ψycf*1, *ndh*F, and *Ψycf*1-*ndh*F non-coding regions located at the IR_B_/SSC junction. Variations in *ndh*C-*ndh*J and *ndh*D-*ndh*H sequences were caused by pseudogenization or loss of *ndh* genes.

**Table 1:** Comparison of major features of seven orchid chloroplast genomes.

Species	*Dendrobium officinale*	*Cymbidium mannii*	*Erycina pusilla*		*Oncidium* Gower Ramsey	*Phalaenopsis Aphrodite*	*Phalaenopsis equestris*	*Cypripedium macranthos*
Subfamily	Epidendroideae							Cypripedioideae
Accession Number	KC771275	KC876129		JF746994	GQ324949	AY916449	JF719062	KF925434
Size (bp)	152,221	155,308		143,164	146,484	148,964	148,959	157,050
LSC length (bp)	85,109	85,212		84,189	82,324	85,957	85,967	85,292
SSC length (bp)	14,516	17,471		12,097	12,650	11,543	11,300	18,285
IR length (bp)	26,298	26,304		23,439	25,755	25,732	25,846	26,777
Total number of different genes	110	113		108	111	110	109	113
Duplicated genes in IR	19	19		19	19	19	19	19
AT content %								
Overall	62.53	63.06		63.35	62.68	64.4	63.35	62.17
Protein-coding gene	61.48	63.05		61.38	61.34	61.97	61.45	61.57
Ribosomal RNA genes	45.34	45.37		45.52	45.43	45.51	45.53	45.21
Untranslated regions	67.00	67.53		68.51	67.40	68.18	68.41	66.10
Gene with introns	18	18		17	18	17	17	18

### Comparison of sequences flanking IR/SC junctions in the Orchidaceae

**Figure 4:**
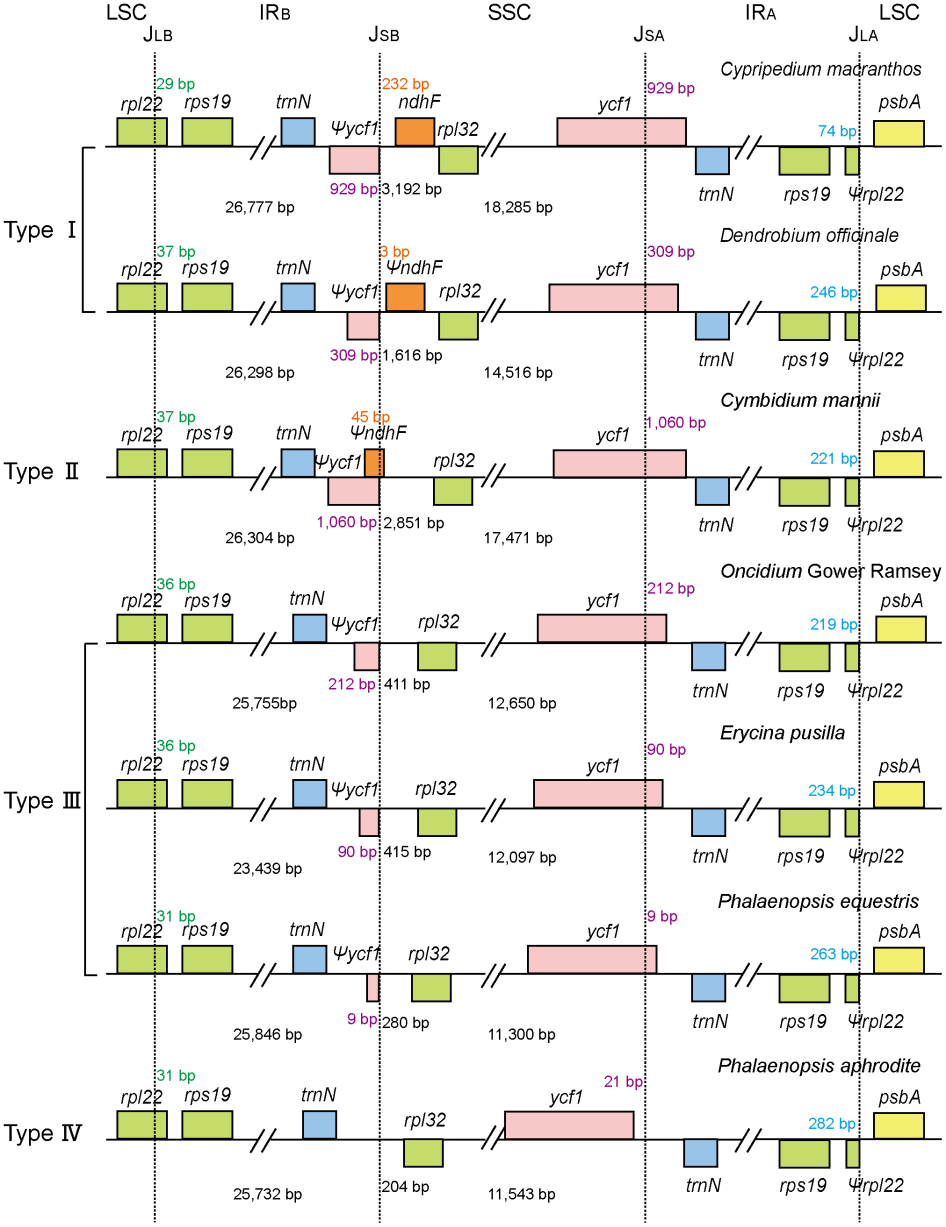
Comparison of the regions flanking the junctions (J_LB_, J_LA_, J_SB_, and J_SA_) among seven orchid chloroplast genomes. Four types of junctions are present at the J_SB_ in seven orchid species. Numbers in green indicate the length of *Ψrpl*22. Numbers in orange indicate the distance between *ndh*F and J_SB_. Numbers in purple indicate the distance between 5′- *ycf*1 and J_SA_. Numbers in blue indicate the distance between *rps*19 and J_LA_. This figure is not to scale.

Sequences flanking IR/SC (single copy) junctions vary among cp genomes of different species [59]. Here, we compared sequences flanking IR/SC junctions among seven orchid cp genomes (Fig. 4); all of them were found to have similar structures at the IR/LSC junction. The *trn*H*-rps*19 cluster was duplicated and involved in IR. The IR_B_/LSC junction (J_LB_) was located within *rpl*22 in all seven orchid cp genomes. As a result, a duplicated *Ψrpl22* was nested within IR_A_.

On the other side, the orchid chloroplast genomes had distinct characteristics at the IR/SSC junction. In *P. Aphrodite*, the IR_A_/SSC junction (J_SA_) was located upstream of *ycf*1, whereas in other species J_SA_ was located within *ycf*1. Four types of junctions in the orchid cp genomes were characterized on the basis of the organization of genes flanking the IR_B_/SSC junction (J_SB_). *Cypripedium* and *Dendrobium* shared type I structure in which J_SB_ was located upstream of the *ndh*F*-rpl*32 cluster. Type II junction was found in *Cymbidium* and was characterized by an overlap between *Ψycf*1 and *ndh*F, resulting in J_SB_ being located within these two genes. Type III was shown in *Oncidium*, *Erycina*, and *P. equestris*, in which J_SB_ was located inside the *Ψycf*1-*rpl*32 cluster, with the loss of *ndh*F gene. The type IV structure was present in *P. aphrodite* and characterized by the entire incorporation of the entire *ycf*1 into the SSC, with J_SB_ inside *trn*N-*rpl*32.

### Chloroplast-encoded *ndh* genes in seven orchid species

Chloroplast-encoded *ndh* genes were investigated in *C. macranthos* and the six photosynthetic Epidendroideae species (Fig. 3). The 11 *ndh* genes in *Cypripedium* cp genome were intact, but many *ndh* genes had either truncations or indels, resulting in frameshifts or pseudogenes in the six Epidendroideae cp genomes. The *ndh*D gene in all these Epidendroideae species contained indels or stop codons. The characteristics of other *ndh* genes differed among the genera. In *Dendrobium*, *ndh*B was intact; *ndh*C, I, and K were lost; and *ndh*F was truncated with two sequence inserts, creating two frameshifts. In the two *Phalaenopsis* species the *ndh*A and *ndh*F genes were absent and the remnants of seven *ndh* genes became pseudogenes. The *ndh*E genes *in P*. *equestris* and *P. aphrodite* were lost and incomplete, respectively. The two Oncidiinae species (*Erycina* and *Oncidium*) had similar patterns of diversity of *ndh* genes except *ndh*A, *ndh*E, and *ndh*I. Other varieties within Oncidiinae shared major characteristics of *ndh* genes in *Erycina* and *Oncidium* [29]. In *Cymbidium*, most of the *ndh* genes were present in the ORF and remained intact [17].

### Sequence divergence of protein-coding genes in the Orchidaceae

The pairwise distances of nucleotide and protein substitutions of 68 protein-coding genes were compared among six orchid species (Table 2). According to the average pairwise distance and numbers of nucleotide substitutions, three genes (*rps*7, *rpl*2, and *rpl*23) located in the IR regions had relatively low mean levels of sequence divergence. The *rpl* and *rps* genes in the LSC and SSC regions showed higher evolutionary rates. Fifteen regions with relatively high divergence were identified in *rps*16, *ycf*1, *mat*K, *rps*15, *rpl*22, *ccs*A, *psa*I, *rpl*32, *rpl*16, *rpl*20, *atp*F, *psb*K, *psb*T, *acc*D, and *rps*8, located in the LSC, SSC, or SSC/IR junction regions. Similar patterns of divergence were also observed at the protein level, with the exception of *psb*T. Sequence divergence and gene length yielded a sufficient variety of loci (>600 bp); thus, the sequences of *acc*D, *ccs*A, *mat*K, and *ycf*1 were identified and used for phylogenetic analyses.

**Table 2:** Pairwise distances of nucleotide and protein substitutions among six orchid species.

Order	Gene	Region	DNA (d)	DNA (S. E.)	Protein (d)	Protein (S.E.)	Miss data	Range of length
1	*rps*7	IR	0.0083	0.0024	0.0065	0.0036		468
2	*rpl*23	IR	0.0085	0.0034	0.0105	0.0078		270–282
3	*rpl*2	IR	0.0140	0.0025	0.0162	0.0052		819–837
4	*pet*G	LSC	0.0150	0.0069	0.0090	0.0086		114
5	*rps*12	IR/LSC	0.0157	0.0036	0.0108	0.0051		372–387
6	*psb*L	LSC	0.0192	0.0078	0.0000	0.0000		117
7	*psb*Z	LSC	0.0234	0.0079	0.0280	0.0140		189
8	*ycf*2	IR	0.0243	0.0013	0.0507	0.0029		6,666–6,876
9	*pet*L	LSC	0.0255	0.0099	0.0323	0.0179		96
10	*psb*F	LSC	0.0255	0.0090	0.0000	0.0000		120
11	*psb*D	LSC	0.0264	0.0030	0.0096	0.0033		1062
12	*atp*H	LSC	0.0269	0.0069	0.0000	0.0000		246
13	*atp*I	LSC	0.0269	0.0039	0.0181	0.0050		744
14	*psa*C	SSC	0.0281	0.0067	0.0000	0.0000		246
15	*psb*E	LSC	0.0291	0.0066	0.0280	0.0114		246–252
16	*psb*N	LSC	0.0298	0.0092	0.0233	0.0141		132
17	*pet*N	LSC	0.0316	0.0136	0.0230	0.0158		90–96
18	*psa*A	LSC	0.0319	0.0025	0.0104	0.0024		2223
19	*ycf*3	LSC	0.0339	0.0051	0.0214	0.0068		507
20	*psb*C	LSC	0.0339	0.0034	0.0038	0.0019		1,224–1,461
21	*rps*19	IR	0.0340	0.0068	0.0313	0.0135	*Cypripedium*	264–279
22	*psa*B	LSC	0.0343	0.0026	0.0135	0.0028		2205
23	*psb*A	LSC	0.0357	0.0034	0.0064	0.0029		1062
24	*pet*A	LSC	0.0360	0.0038	0.0307	0.0058		963
25	*clp*P	LSC	0.0380	0.0049	0.0325	0.0070		594–615
26	*pet*D	LSC	0.0383	0.0056	0.0231	0.0084		531–564
27	*pet*B	LSC	0.0390	0.0046	0.0149	0.0045		648–654
28	*rpo*C2	LSC	0.0396	0.0021	0.0798	0.0047		4,137–4,167
29	*rbc*L	LSC	0.0398	0.0038	0.0243	0.0049		1,443–1,473
30	*atp*A	LSC	0.0416	0.0031	0.0333	0.0048		1,524–1,530
31	*atp*B	LSC	0.0419	0.0036	0.0311	0.0049		1,488–1,497
32	*inf*A	LSC	0.0424	0.0081	0.0052	0.0049	*Cypripedium*	
33	*rps*4	LSC	0.0426	0.0047	0.0544	0.0102		606
34	*ycf*4	LSC	0.0432	0.0056	0.0500	0.0102		555
35	*psb*B	LSC	0.0451	0.0037	0.0143	0.0034		1,527
36	*psb*I	LSC	0.0466	0.0125	0.0000	0.0000		111
37	*rpl1*4	LSC	0.0467	0.0073	0.0399	0.0107		369
38	*rpo*B	LSC	0.0474	0.0041	0.0474	0.0041		3213
39	*rps*18	LSC	0.0479	0.0079	0.0429	0.0112		306–315
40	*rps*14	LSC	0.0487	0.0081	0.0620	0.0157		303
41	*rpo*C1	LSC	0.0493	0.0032	0.0559	0.0053		2,034–2,061
42	*rpl*36	LSC	0.0521	0.0143	0.0270	0.0150		114
43	*psb*J	LSC	0.0556	0.0146	0.0350	0.0192		123
44	*atp*E	LSC	0.0569	0.0079	0.0687	0.0141		402–405
45	*cem*A	LSC	0.0573	0.0063	0.0749	0.0104		687–690
46	*rps*2	LSC	0.0573	0.0057	0.0599	0.0092		711
47	*psa*J	LSC	0.0573	0.0127	0.0286	0.0164		129–135
48	*psb*M	LSC	0.0578	0.0165	0.0196	0.0135		105–114
49	*rpo*A	LSC	0.0609	0.0049	0.0956	0.0095		1,014–1,020
50	*rps*11	LSC	0.0616	0.0076	0.0599	0.0124		415
51	*rpl*33	LSC	0.0623	0.0118	0.1030	0.0193		201
52	*psb*H	LSC	0.0652	0.0113	0.0831	0.0218		222
53	*rps*3	LSC	0.0656	0.0066	0.0799	0.0109		648–663
54	*rps*8	LSC	0.0674	0.0082	0.0824	0.0155		396–399
55	*acc*D	LSC	0.0681	0.0044	0.1187	0.0088		1,449–1,491
56	*psb*T	LSC	0.0681	0.0185	0.0101	0.0097		102–111
57	*psb*K	LSC	0.0688	0.0132	0.0918	0.0241		186
58	*atp*F	LSC	0.0701	0.0070	0.0894	0.0119		549–555
59	*rpl*20	LSC	0.0794	0.0100	0.0963	0.0170		354–375
60	*rpl*16	LSC	0.0796	0.0083	0.0662	0.0122		396–411
61	*rpl*32	SSC	0.0808	0.0150	0.1079	0.0276		171–177
62	*psa*I	LSC	0.0886	0.0192	0.0796	0.0280		111–189
63	*ccs*A	SSC	0.0916	0.0065	0.1235	0.0116		966–996
64	*rpl*22	IR/LSC	0.0969	0.0104	0.1174	0.0184		360–405
65	*rps*15	SSC	0.1015	0.0132	0.1408	0.0215		270–276
66	*mat*K	LSC	0.1033	0.0063	0.1613	0.0098		1,404–1,566
67	*ycf*1	IR/SSC	0.1477	0.0039	0.2448	0.0076	*Phalaenopsis*, *Oncidium*	5,307–5,520
68	*rps*16	LSC	0.3001	0.0295	0.2928	0.0291	*Cymbidium*	279–308

‘d’ and ‘S.E.’ indicate overall mean distances and standard errors, respectively. Nucleotide and amino acid distances were calculated using Kimura's 2-parameter model distances and a p-distance model, respectively.

### Molecular phylogeny within the Epidendroideae

To determine the availability of the *acc*D, *ccs*A, *mat*K, and *ycf*1 sequences for phylogenetic analyses, the Epidendroideae was used as a case study because of the disputes regarding its systematics. Sequences of the four genes were successfully amplified in all 46 taxa. The aligned combined dataset comprised 4,593 characters, of which 2,839 represented variable sites and 1,447 were parsimony-informative sites. The number of variable sites was highest in *ycf*1 and lowest in *ccs*A (Table S6).

**Figure 5:**
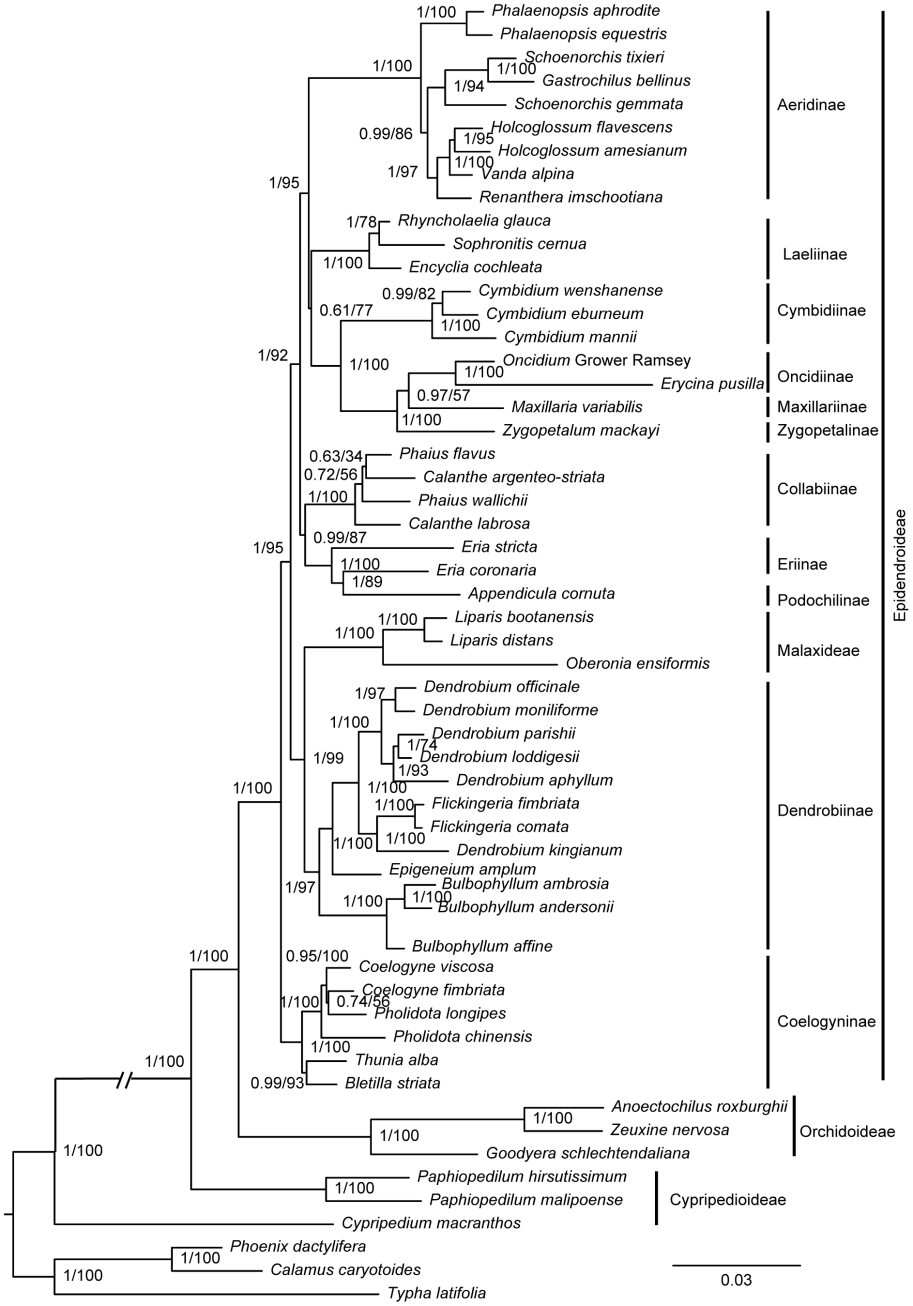
Phylogenetic tree of the Epidendroideae reconstructed based on combined genes. BI and ML analyses yielded identical topologies. Posterior probability and bootstrap proportion are indicated near the nodes. Subfamilies, tribes, and subtribes (sensu Chase et al. [2]) are indicated where applicable.

Phylogenetic analyses using BI and ML approaches resulted in the same topology (Fig. 5). Most nodes had high support among tribes and subtribes within the subfamily Epidendroideae. Within it, Coelogyninae was sister to all the other subtribes or tribes, with strong support (ML BP 100%, BI PP 1.00). The *Bulbophyllum* group clustered with the *Epigeneium* and *Dendrobium*-*Flickingeria* to form a monophyletic clade of Dendrobiinae that was closely allied to Malaxideae (*Laparis* and *Oberonia*). The Dendrobiinae-Malaxideae clade was sister to the rest of the subfamily. Podochilinae and Eriinae were not monophyletic clades; these two subtribes (both of tribe Podochileae) were sister to Collabiinae.

## Discussion

### Comparison of RNA editing sites

Involved in plastid posttranscriptional regulation, RNA editing provides an effective way to create transcript and protein diversity [60], [61]. Some chloroplast RNA editing sites are conserved in higher plants [62], [63]. In Orchidaceae, RNA editing sites were identified in 24 protein-coding transcripts in *P. aphrodite* [63]. Potential editing sites also were identified in *P. equestris* and *O*. Gower Ramsey [33]. Of the examined 30 genes in above-mentioned seven orchids, 15 potential RNA editing sites out of 11 genes (*atp*A, *atp*F, *clp*P, *mat*K, *pet*B, *psb*F, *rpl*20, *rpo*A, *rpo*B, *rpo*C1 and *ycf*3) were shared; the number of shared editing sites increased in Epidendroideae species (28 sites out of 16 genes) (Table S5 and [33]). Therefore, RNA editing is more conserved from the same subfamily than which from different subfamily. However, orchids and other angiosperms have relatively less common editing sites. For example, 10 potential RNA editing sites were shared by orchids and *Cocos nucifera*; comparisons among *Nicotiana tabacum*, *Arabidopsis thaliana*, grasses and orchid RNA editing sites showed low conservation of editing sites (only one common editing sites in *rpo*B)(Table S5). These cases indicate that the evolutionary conservation of RNA editing is essential for only a few plastid-editing sites [64]–[66].

### IR expansion or contraction in the Orchidaceae

The variability of genes flanking IR/SC junctions results in IR expansion or contraction [59], [67]. At the IR/LSC boundaries, most IRs of non-orchid monocots contained *trn*H-*rps*19 gene clusters, excluding *Ψrpl*22 genes, leading to more-progressive expansion of IRs than that having occurred in non-monocot angiosperms [17], [20], [55], [59], [68]–[71]. In all known photosynthetic orchid cp genomes, *trn*H-*rps*19 clusters and *Ψrpl*22 genes were involved in IRs at the IR/LSC junctions. The IR/LSC junctions were the standard type III [71], and IRs experienced the largest expansion at the IR/LSC junction compared with other monocots.

The IR/SSC junction types II–IV in orchids differed from those in other monocots, while type I (in *Cypripedium* and *Dendrobium* cp genomes) was similar to that in *Acorus* (Fig. 4) with *ycf*1 extending over the J_SA_ and*Ψycf*1 located within IR adjacent to the J_SB_. Although Yang et al. suggested most likely evolutionary routes of IRs in monocots [59], no studies have proposed a model about the evolutionary dynamics of the IR/SSC junctions within orchids. Here, we hypothesize two evolutionary routes to explain the expansion or contraction of IRs adjacent to IR/SSC junctions from an *Acorus*-like ancestor to the existing orchids. The first route proceeded from type I to type II; *ycf*1 further expanded into the IR_A_, resulting in an expansion of duplicated *Ψycf*1 in the IR_B_. During this period, an overlap occurred between *ndh*F remnant and *Ψycf*1. On the second route, *ycf*1 shifted continuously into the SSC, resulting in a shorter, duplicated *Ψycf*1 adjacent to the J_SB_. Continually, *ycf*1 was embedded completely into the SSC, leading to the loss of duplicated *Ψycf*1. This contractive process of IR involved the structural change from type I to type IV via type III. Moreover, IRs expansion or contraction may not correlate with the taxonomic relationships. More molecular data need to be collected for intensifying our understanding of variations in sequences flanking IR/SSC junctions.

The shift of the border between the IR and SSC in orchids was associated with the *ycf*1 gene. Compared with the average AT content of protein-encoding genes, all known orchid *ycf*1 genes exhibited usage bias of AT base pairs (see Table 1 and Table S7). AT base pairs are bound by two hydrogen bonds, while GC base pairs are bound by three hydrogen bonds; therefore, DNA with high AT content is less stable than that with low AT content. Poly (A) tract sequences at IR/LSC boundaries might be closely linked with the dynamics of IR/LSC junctions and expansion of IR [67], [71]. Similarly, the AT-rich nature of *ycf*1 gene may be linked to the recombination of IR/SSC junction.

### The loss or pseudogenization of *ndh* genes in orchid chloroplast genomes

Instances of gene loss or pseudogenes have been elucidated in the cp genomes of monocots [21]. Chloroplast-encoded gene degeneration in photosynthetic orchids is mostly embodied in structural changes of *ndh* genes. There are 11 chloroplast-encoded *ndh* genes in the cp genomes of land plants, located in several transcriptional units and encoding for the thylakoid Ndh complex [72]. Non-functional chloroplast-encoded *ndh* genes have been found in CAM and C3 plants [32], including gymnosperms and grasses [68], [73], [74]. Sequence truncations and indels are common phenomena in orchid chloroplast-encoded *ndh* genes [17], [28]–[33], [75]. Pseudogenization or loss of the *ndh* gene did not correlate well with the divergent patterns of Epidendroideae lineages observed in the phylogenetic trees (Fig. 3). However, 10 common *ndh* pseudogenes of two *Phalaenopsis* species showed a high degree of similarities in sequence and indel patterns [33]. Both *Erycina* and the allied genus *Oncidium* lost two *ndh* genes (*ndh*F and *ndh*K) and had six pseudogenes (*ndh*B, C, D, G, and J); similar results were obtained from *Oncidium* and related Oncidiinae varieties [29]. Thus, we infer that relative species had similar patterns of variation in *ndh* gene content.

The loss of some chloroplast-encoded genes might not affect the plant life cycle. Gene transfer from chloroplast to nucleus is known to occur frequently during evolutionary processes [76]. The ancestral plastid *ndh* genes of orchids are presumed to have been transferred to the nucleus [28]. Moreover, fungal symbionts may contribute to the fate of *ndh* genes [77]. Therefore, the functions of lost chloroplast-encoded *ndh* genes could be performed by homologous genes from other resources; this hypothesis needs to be tested in the future study.

### Phylogenetic relationships based on complete cp genomes

The cp sequences have been used in deep phylogenetic analyses because of their low substitution rates [20], [78]. Phylogenetic analyses based on complete chloroplast genomes have resolved some bewildering relationships in angiosperms. Using two tree construction methods with different models, we obtained consistent results on the relationships among *Phalaenopsis* (Aeridinae), *Cymbidium* (Cymbidiinae), *Dendrobium* (Dendrobiinae), *Oncidium* and *Eryc*ina (Oncidiinae) within Epidendroideae, which are congruent with *mat*K and *rbc*L analyses by Gustafsson et al. (2010) [8] and morphological cladistic analysis by Freudenstein and Rasmussen (1999) [12]; but are inconsistent with the analyses based on nuclear ribosomal internal transcribed spacer (nrITS), *mat*K, *rbc*L, *trn*L-F, the *trn*L intron, and nuclear *Xdh* gene [4], [7]. However, whole-genome sequencing for sparse sampling can result in long-branch artifacts and incorrect evolutionary reconstructions [79]. Therefore, further genomic and taxon sampling will be necessary to resolve the relationships within this subfamily.

### Gene divergence based on comparative chloroplast genomes

Variability of genes in cp genomes has been calculated according to nucleotide diversity in previous studies [80], [81]. If we considered sequence divergence at the nucleotide and protein levels, *rps*7, *rpl*23, *rpl*2, and *ycf*2 were conserved with low evolutionary distance, with the exception of *rps*19, which exhibited medium divergence in the IR regions. These results are consistent with previous reports of slower divergence of sequences in the IR regions compared to other regions [80], [82]. Although the *ycf*2 gene has been demonstrated to be one of the most rapidly evolved genes among 16 vascular plant species [80], the present study showed that it had relatively slow nucleotide divergence and moderate protein divergence within the Orchidaceae.

In this study, highly divergent genes were acquired according to pairwise distance of nucleotide substitutions. While *ycf*1 was located at the IR/SSC junction, 14 other genes bordered the LSC and SSC regions, four of which were selected to construct phylogenetic trees. Of these, *mat*K and *ycf*1 have been used in previous studies [4], [6], while *acc*D and *ccs*A were applied for the first time to the phylogenetic analysis of the subfamily Epidendroideae in the present study. These genes can be used as good phylogenetic markers at the subfamily level because of the following three reasons. First, these regions are variable, which highlights their unusual evolutionary properties. According to the pairwise distance of protein substitutions (Table 2), *ycf*1 and *mat*K have high divergence, and a*cc*D and *ccs*A have relatively moderate substitution rates that are higher than *rbc*L, which has been used in previous systematic analyses within the Epidendroideae [4], [5], [75]. Second, these regions are sufficiently long (>600 bp) to yield adequate loci for phylogenetic analysis. Third, the sequences are easily obtained by PCR amplification and relatively conservative for alignment.

### Phylogenetic reconstruction of the Epidendroideae

The phylogeny of the Epidendroideae has long been debated. Here, eleven common subtribes and one tribe from Epidendroideae were used as a case study to identify the phylogenetic relationships within this subfamily using four cp sequences. Fig. S1 illustrates the relationships among these subtribes or tribes in previous studies based on molecular data. With polyphyletic and paraphyletic groups excluded from phylogenetic analyses, major debates were the placement of Dendrobiinae, Malaxideae, and Collabiinae, as well as identification of the basal subtribe or tribe. On the basis of a concatenated data set, we clarified several relationships that were previously poorly resolved, and the majority of nodes at the subtribe level in the gene trees had high support.

The placement of Coelogyninae (tribe Arethuseae) varied according to morphological and molecular proofs. Based on observing that Arethuseae has cormous and reed-stem habits, Dressler (1986) claimed that Arethuseae is the basal group in the “reed-stem” phylad [83]. Dressler (1990) divided advanced Epidendroideae into four major clades (Gastrodieae, Nerviieae, Cymbidioid phylad, and Epidendroid phylad); and placed Arethuseae and Dendrobioid subclade in Epidendroid phylad, and Maxillarieae, Cymbidieae, Malaxideae in Cymbidioid phylad [13]. Dressler (1993) held that Arethuseae appeared to be paraphyletic due to their ever-shifting boundaries and tenuous morphological definitions [84]. However, Van den Berg et al. (2005) fixed subtribe Coelogyninae in distinct positions based on different methods using nrITS and four plastid sequences [4]. The results in this study strongly support that Coelogyninae was the most basal subtribe within the sampled subtribes, which was in line with the BI analysis by Van den Berg et al. (2005) [4], the MP analysis by Neubig et al. (2009) [6], and analyses by Gorniak et al. (2010) [7].

Previously, the placement of Collabiinae and Dendrobiinae was problematic, but their positions have been recovered. Collabiinae was polyphyletic based on *mat*K and *rbc*L [15]. Van Den Berg (2005) proposed Collabiinae was in an unfixed position in MP and BI analyses, and Gorniak et al. (2010) posited that Collabiinae was sister to Aeridinae and Eriinae with high support based on nuclear gene *Xdh* [4], [7]. Our results suggest that Collabiinae was sister to the Podochilinae-Eriinae (tribe Podochileae) clade with moderate support; this is congruent with MP analysis of Van Den Berg (2005), which had weak support [4]. The positions of Dendrobiinae and Malaxideae were also confirmed. Dressler (1990) placed Malaxideae and Dendrobieae in two separated groups, Cymbidioid phylad and Epidendroid phylad, according to reed stem, upper lateral inflorescences and spherical silica bodies [13]. Chase (2003) recognized Dendrobiinae as a subtribe rather than tribe Dendrobieae [2]. By inferring from nrITS and four chloroplast sequences, Van den Berg et al. (2005) held that Dendrobiinae was beside Malaxideae [4]. Dendrobiinae is similar to Malaxideae in the synapomorphic state of the naked pollinium [84]. Like other analyses based on *Xdh* and *rbc*L, our analyses support that Dendrobiinae and Malaxideae were sister relatives [5], [7], which was consistent with the morphological similarities between them. Controversially, the position of Dendrobiinae-Malaxideae clade was going up in the analysis of *Xdh* [7], and Podochileae was sister to this clade in the analysis of plastid gene *rbc*L (bootstrap support <50%) [5]; however, this clade was sister to other clades except Coelogyninae with high support in the present study. More extensive sampling and sequencing of mitochondrial and nuclear genomes should be conducted to resolve uncertain relationships within the Epidendroideae with confidence.

## Conclusions

In summary, complete chloroplast genomes can provide abundant information for resolving evolutionary questions. The gene content, organization, and sequence of chloroplast genome have been used as important markers in systematic research. This study determined complete cp genomes of *Dendrobium officinale* and *Cypripedium macranthos* and compared cp genomes of seven photosynthetic orchids including the above two, which showed structural similarities but differences in IR/SSC junctions and *ndh* genes. We propose that the AT bias of *ycf*1 in the Epidendroideae may be related to recombination of the IR/SSC junction. In addition, relationships among subtribes and tribes in the subfamily Epidendroideae were resolved with high or moderate support in the present study. The highly divergent genes of cp genomes identified in this study can be used as markers in phylogenetic analyses. Further plastome sequencing of orchids will be necessary to clarify the diversity of chloroplast genomes and to improve our understanding of the relationships within this family.

## Supporting Information

Figure S1 **Phylogenetic relationships among 11 subtribes and one tribe within the subfamily Epidendroideae resulting from previous studies.** All trees were drawn according to the cited references. Molecular markers and methods used in phylogenetic analyses are enclosed by parentheses below the cited studies. Subtribal and tribal delimitations refer to Chase et al. [2].
(TIF)

Table S1 **Primers used for gap closure, assembly and junction verification.**
(DOC)

Table S2 **Accession numbers for taxa used in phylogenomic analysis and genome comparison.**
(DOC)

Table S3 **Primers for phylogenetic analyses of orchids.**
(DOC)

Table S4 **Taxa and NCBI accession numbers used in phylogenetic analyses of the Epidendroideae.**
(DOC)

Table S5 **RNA editing predicted in** ***Dendrobium officinale*** **and** ***Cypripedium macranthos*** **chloroplast genomes by the PREP program.**
(DOC)

Table S6 **Sequence information for genes used in the phylogenetic analysis of the Epidendroideae.**
(DOC)

Table S7 **AT content of the** ***ycf*** **1 gene in the Orchidaceae.**
(DOC)
