# Supplementary material for: Comparative Chloroplast Genomes of Photosynthetic Orchids: Insights into Evolution of the Orchidaceae and Development of Molecular Markers for Phylogenetic Applications
Source: PLoS One. 2014 Jun 9;9(6):e99016. doi: 10.1371/journal.pone.0099016 (PMC4049609; doi:10.1371/journal.pone.0099016)
Supplement: Table S1 — Primers used for gap closure, assembly and junction verification. (DOC) [file pone.0099016.s002.doc]

Table S1. Primers used for gap closure, assembly and junction verification.

| Primer no. | Forward primer sequence (5’→3’) | Reverse primer sequence (5’→3’) |
| --- | --- | --- |
| D1 | TGTAGCTCAGAGGATTAGAGCACGT | TTCTCTTTGTGGTATTCCGCCTCTT |
| D2 | TAATCTGGCTAACGTGGAACTTGG | TTAAACTCTACTGCGGTGACGATAC |
| D3 | CCGACTAGTTCCGGGTTCGA | ACGGGAATTGAACCCGCGCA |
| D4 | TAGCGCGAATTGTTTGTAGGG | CGAACGACGGGAATTGAACC |
| D5 | CGGACGGGAAGTGGTGTTT | GCTTCATGCAGGCGAGTTG |
| D6 | TATCAGATAACTACGTCCTCC | CGCGCATGGTGGATTCACAAATC |
| D7 | CTACCACTGAGCTACTGAGGAACAAC | TGACTGTCCGGTGGAAACAGATTTAG |
| D8 | TGTAGCTCAGAGGATTAGAGCACGT | AGAAGCAATGACGGTATCTGAGGAATA |
| D9 | CTTCATAGGGTCTTTCTGTCCAGGT | TAACGGTCCTAAGGTAGCGAAATTCC |
| D10 | TTGGCTGTTAACTGACTGGTCGTAG | GGGCTGTAGTATGTTCCAAGGGTTG |
| D11 | TAATCTGGCTAACGTGGAACTTGG | TTAAACTCTACTGCGGTGACGATAC |
| C1 | CTTCTGCTTCTGGTTGTCT | CTTGGTTCTTGGTTGAGGA |
| C2 | CTAATCGCTCGAATCTTTGTTG | CGCCATAGAAGGACTAGGAAA |
| C3 | ATTTGTGACCTACCATACGA | ACTGCCTTGATCCACTTG |
| C4 | GTCTCCGTATCTTACTGGTG | GTCTATCTATACGAAGGGACGG |
| C5 | CATTACAAATGCGATGCTCT | TCTACCGATTTCGCCATATC |
| C6 | TTGTTGCTGATACCTCCTTC | CGAACCTACGACCAGTCA |
| C7 | TACCGCCTGTGAATACTC | GTCGTGGATACTGCTGTA |
| C8 | CCGAATACTCTACCGTTGA | TGAAACGATGTGGTAGAAAG |
| C9 | CCGAATACTCTACCGTTGA | TGTTGTTGTGCCAATCCA |
| C10 | AATCCAGCAAACCCATCAT | TCATTTATCGGGAGGAAGAA |

Note: D1-11 were used for *Dendrobium officinale* chloroplast genome and C1-10 were used for *Cypripedium macranthos* chloroplast genome.
