# Supplementary material for: Comparative Chloroplast Genomes of Photosynthetic Orchids: Insights into Evolution of the Orchidaceae and Development of Molecular Markers for Phylogenetic Applications
Source: PLoS One. 2014 Jun 9;9(6):e99016. doi: 10.1371/journal.pone.0099016 (PMC4049609; doi:10.1371/journal.pone.0099016)
Supplement: Table S2 — Accession numbers for taxa used in phylogenomic analysis and genome comparison. (DOC) [file pone.0099016.s003.doc]

**Table S2. Accession numbers for taxa used in phylogenomic analysis and genome comparison.**

| **Taxon** | **Family** | **Accession number** |
| --- | --- | --- |
| *Calamus caryotoides* A. Cunn ex Mart. | Arecaceae | JX088663 |
| *Phoenix dactylifera* L. | Arecaceae | GU811709 |
| *Typha latifolia* L. | Typhaceae | GU195652 |
| *Cypripedium macranthos* Sw. | Orchidaceae | KF925434 |
| *Rhizanthella gardneri* R. S. Rogers | Orchidaceae | GQ413967 |
| *Corallorhiza striata* var. *vreelandii* | Orchidaceae | JX087681 |
| *Cymbidium mannii* H. G. Reichenbach | Orchidaceae | KC876126 |
| *Dendrobium officinale* Kimura et Migo | Orchidaceae | KC771275 |
| *Erycina pusilla* (L.) N.H.Williams & M.W.Chase | Orchidaceae | JF746994 |
| *Neottia nidus-avis* (L.) L. C. Rich. | Orchidaceae | JF325876 |
| *Oncidium* Grower Ramsey | Orchidaceae | GQ324949 |
| *Phalaenopsis* *aphrodite* Reichb. f. | Orchidaceae | AY916449 |
| *Phalaenopsis equestris* (Schauer) Rchb. | Orchidaceae | JF719062 |
