# Supplementary material for: Comparative Chloroplast Genomes of Photosynthetic Orchids: Insights into Evolution of the Orchidaceae and Development of Molecular Markers for Phylogenetic Applications
Source: PLoS One. 2014 Jun 9;9(6):e99016. doi: 10.1371/journal.pone.0099016 (PMC4049609; doi:10.1371/journal.pone.0099016)
Supplement: Table S3 — Primers for phylogenetic analyses of orchids. (DOC) [file pone.0099016.s004.doc]

**Table S3. Primers for phylogenetic** **analyses of orchids.**

| **Genes** | **Primer names** | **Primer sequences (5’→3’)** | **Sources** |
| --- | --- | --- | --- |
| *ycf*1 | ycf1_3720F | TACGTATGTAATGAACGAATGG | [1] |
|  | ycf1_5500R | GCTGTTATTGGCATCAAACCAATAGCG | [1] |
| *acc*D | accD_F | TGGATAGTCTTGATGCTCTTG | This study |
|  | accD_R | GTTGTAGGAGATGTAAGGATTG | This study |
| *mat*K | 390F | CGATCTATTCATTCAATATTTC | [2] |
|  | 1326R | TCTAGCACACGAAAGTCGAAGT | [2] |
| *ccs*A | ccsA-F | CGTTGGATTTCTTCGGGACAT | This study |
|  | ccsA-R | AGTTTCCTTAGGGTCCCAATT | This study |

**References**

1. Neubig KM, Whitten WM, Carlsward BS, Blanco MA, Endara L, et al. (2009) Phylogenetic utility of *ycf*1 in orchids: a plastid gene more variable than *mat*K. Plant Syst Evol 277: 75-84.
2. Lahaye R, Van der Bank M, Bogarin D, Warner J, Pupulin F, et al. (2008) DNA barcoding the floras of biodiversity hotspots. Proc Natl Acad Sci USA 105: 2923-2928.
