# Supplementary material for: Comparative Chloroplast Genomes of Photosynthetic Orchids: Insights into Evolution of the Orchidaceae and Development of Molecular Markers for Phylogenetic Applications
Source: PLoS One. 2014 Jun 9;9(6):e99016. doi: 10.1371/journal.pone.0099016 (PMC4049609; doi:10.1371/journal.pone.0099016)
Supplement: Table S4 — Taxa and NCBI accession numbers used in phylogenetic analyses of the Epidendroideae. (DOC) [file pone.0099016.s005.doc]

**Table S4. Taxa and NCBI accession numbers used in phylogenetic analyses of the Epidendroideae**.

| **Family, subfamily, tribe, subtribe,** **genus and species** | | **Sources/Vouchers** | **GenBank accession numbers** | | | | |
| --- | --- | --- | --- | --- | --- | --- | --- |
|  |  |  | ***acc*D** | ***ccs*A** | ***mat*K** | ***ycf*1** | |
| **Orchidaceae** | | | | | | | |
| **Epidendroideae** | | | | | | | |
| Arethuseae |  |  |  |  |  |  | |
| Coelogyninae |  |  |  |  |  |  | |
|  | *Coelogyne fimbriata* Lindl. | Yunnan, China/ECoeF | KF361527 | KF361573 | KF361619 | KF361665 | |
|  | *Bletilla striata* (Thunb. ex A. Murray) Rchb. f. | Yunnan, China/Ble26 | KF361563 | KF361609 | KF361655 | KF361701 | |
|  | *Thunia alba* (Lindl.) Rchb. f. | Yunnan, China/EThuA | KF361537 | KF361583 | KF361629 | KF361675 | |
|  | *Coelogyne viscosa* Rchb. f. | Yunnan, China/ECoeV | KF361542 | KF361588 | KF361634 | KF361680 | |
|  | *Pholidota chinensis* Lindl. | Yunnan, China/EPhoC | KF361543 | KF361589 | KF361635 | KF361681 | |
|  | *Pholidota longipes* S. C. Chen & Z. H. Tsi. | Yunnan, China/EPhoL | KF361548 | KF361594 | KF361640 | KF361686 | |
| Cymbidieae |  |  |  |  |  |  | |
| Cymbidiinae |  |  |  |  |  |  | |
|  | *Cymbidium eburneum* Lindl. | Yunnan, China/ECynE | KF361558 | KF361604 | KF361650 | KF361696 | |
|  | *Cymbidium wenshanense* Y. S. Wu & F. Y. Liu | Yunnan, China/ECymW | KF361554 | KF361600 | KF361646 | KF361692 | |
|  | *Cymbidium mannii* Rchb. f. | [1] | KC876126 | | | | |
| Oncidiinae |  |  |  |  |  | |  |
|  | *Oncidium* Grower Ramsey | [2] | GQ324949 | | | | |
|  | *Erycina pusilla* (L.) N. H. Williams & M. W. Chase | [3] | JF746994 | | | | |
| Epidendreae |  |  |  |  |  | |  |
| Laeliinae |  |  |  |  |  | |  |
|  | *Rhyncholaelia glauca* (Lindl.) Schltr. | Yunnan, China/ERhyG | KF361555 | KF361601 | KF361647 | | KF361693 |
|  | *Encyclia cochleata* (L.) Dressler | Guangdong, China/EENcC | KF361551 | KF361597 | KF361643 | | KF361689 |
|  | *Sophronitis cernua* Lindl. | Brazil/ESopC | KF361553 | KF361599 | KF361645 | | KF361691 |
| Podochileae |  |  |  |  |  | |  |
| Eriinae |  |  |  |  |  | |  |
|  | *Eria coronaria* (Lindl.) Rchb. f. | Yunnan, China/Eeric | KF361549 | KF361595 | KF361641 | | KF361687 |
|  | *Eria stricta* Lindl. | Yunnan, China/EEriN | KF361557 | KF361603 | KF361649 | | KF361695 |
| Podochilinae | |  |  |  |  | |  |
|  | *Appendicula cornuta* Bl. | Yunnan, China/EAppC | KF361559 | KF361605 | KF361651 | | KF361697 |
| Vandeae |  |  |  |  |  | |  |
| Aeridinae |  |  |  |  |  | |  |
|  | *Renanthera imschootiana* Rolfe | Yunnan, China/ERenI | KF361529 | KF361575 | KF361621 | | KF361667 |
|  | *Schoenorchis tixieri* (Guillaum.) Seidenf. | Yunnan, China/ESchT | KF361528 | KF361574 | KF361620 | | KF361666 |
|  | *Schoenorchis gemmata* (Lindl.) J. J. Smith | Yunnan, China/ESchG | KF361534 | KF361580 | KF361626 | | KF361672 |
|  | *Holcoglossum amesianum* (Rchb. f.) Christenson | Yunnan, China/Ehola | KF361540 | KF361586 | KF361632 | | KF361678 |
|  | *Holcoglossum flavescens* (Schltr. ) Z. H. Tsi | Yunnan, China/EHolF | KF361531 | KF361577 | KF361623 | | KF361669 |
|  | *Vanda alpina* Lindl. | Yunnan, China/EVanA | KF361539 | KF361585 | KF361631 | | KF361677 |
|  | *Gastrochilus bellinus* (Rchb. f.) Kuntze | Yunnan, China/EGasB | KF361532 | KF361578 | KF361624 | | KF361670 |
|  | *Phalaenopsis* *aphrodite* Reichb. f. | [4] | AY916449 | | | | |
|  | *Phalaenopsis equestris* (Schauer) Rchb.f. | [5] | JF719062 | | | | |
| Malaxideae |  |  |  |  |  | |  |
|  | *Liparis distans* C. B. Clarke | Yunnan, China/ELipD | KF361535 | KF361581 | KF361627 | | KF361673 |
|  | *Liparis bootanensis* Griff. var. bootanensis | Guangxi, China/E;ipB | KF361545 | KF361591 | KF361637 | | KF361683 |
|  | *Oberonia ensiformis* (J. E. Smith) Lindl. | Guangxi, China/EObeE | KF361567 | KF361613 | KF361659 | | KF361705 |
| Maxillarieae |  |  |  |  |  | |  |
| Maxillariinae |  |  |  |  |  | |  |
|  | *Maxillaria variabilis* Bateman ex Lindl. | Yunnan, China/EMaxV | KF361533 | KF361579 | KF361625 | | KF361671 |
| Zygopetalinae |  |  |  |  |  | |  |
|  | *Zygopetalum mackayi* Hook. | Yunnan, China/EzygM | KF361552 | KF361598 | KF361644 | | KF361690 |
| Epidendroideae incertae sedis | |  |  |  |  | |  |
| Collabiinae |  |  |  |  |  | |  |
|  | *Calanthe labrosa* (Rchb. f.) Rchb. f. | Yunnan, China/ECalL | KF361538 | KF361584 | KF361630 | | KF361676 |
|  | *Calanthe argenteo*-*striata* C. Z. Tang & S. J. Cheng | Guangxi, China/ECalA | KF361544 | KF361590 | KF361636 | | KF361682 |
|  | *Phaius flavus* (Bl.) Lindl. | Yunnan, China/EPhaF | KF361536 | KF361582 | KF361628 | | KF361674 |
|  | *Phaius wallichii* Lindl. | Yunnan, China/EPhaW | KF361541 | KF361587 | KF361633 | | KF361679 |
| Dendrobiinae | |  |  |  |  | |  |
|  | *Bulbophyllum ambrosia* (Hance) Schltr. | Yunnan, China/Bam | KF361565 | KF361611 | KF361657 | | KF361703 |
|  | *Bulbophyllum andersonii* (Hooker. f.) J. J. Smith | Yunnan, China/EBulA | KF361550 | KF361596 | KF361642 | | KF361688 |
|  | *Bulbophyllum affine* Lindl. | Yunnan, China/Baf | KF361566 | KF361612 | KF361658 | | KF361704 |
|  | *Dendrobium aphyllum* (Roxb.) C. E. C. Fisch. | Yunnan, China/D3 | KF361568 | KF361614 | KF361660 | | KF361706 |
|  | *Dendrobium parishii* Rchb. f. | Yunnan, China/D58 | KF361524 | KF361570 | KF361616 | | KF361662 |
|  | *Dendrobium kingianum* Bidwill ex Lindl. | Australia/D80 | KF361569 | KF361615 | KF361661 | | KF361707 |
|  | *Dendrobium officinale* Kimura et Migo | Zhejiang, China/Denoff | KC771275 | | | | |
|  | *Dendrobium loddigesii* Rolfe | Yunnan, China/Dlo-2 | KF361525 | KF361571 | KF361617 | | KF361663 |
|  | *Dendrobium moniliforme* (L.) Sw. | Yunnan, China/Dmo-y | KF361526 | KF361572 | KF361618 | | KF361664 |
|  | *Epigeneium amplum* (Lindl.) Summerh. | Yunnan, China/EEpiA | KF361530 | KF361576 | KF361622 | | KF361668 |
|  | *Flickingeria comata* (Bl.) Hawkes | Guangxi, China/F_co | KF361546 | KF361592 | KF361638 | | KF361684 |
|  | *Flickingeria fimbriata* (Bl.) Hawkes | Guangxi, China/F_fi | KF361547 | KF361593 | KF361639 | | KF361685 |
| **Cypripedioideae** | | | | | | | |
|  | *Paphiopedilum malipoense* S. C. Chen et Z. H. Tsi | Guangdong, China/PapM | KF361561 | KF361607 | KF361653 | | KF361699 |
|  | *Paphiopedilum hirsutissimum* (Lindl. ex Hook.) Stein | Guangdong, China/PapH | KF361560 | KF361606 | KF361652 | | KF361698 |
|  | *Cypripedium macranthos* Sw. | Yunnan, China/Cypma | KF925434 | | | | |
| **Orchidoideae** | | | | | | | |
| Cranichideae | |  |  |  |  | |  |
|  | *Anoectochilus roxburghii* (Wall.) Lindl. | Guangdong, China/EAneR | KF361564 | KF361610 | KF361656 | | KF361702 |
|  | *Goodyera schlechtendaliana* Rchb. f. | Guangdong, China/GooS | KF361562 | KF361608 | KF361654 | | KF361700 |
|  | *Zeuxine nervosa* (Lindl.) Trimen | Yunnan, China/EZenN | KF361556 | KF361602 | KF361648 | | KF361694 |
| **Outgroup** | | | | | | | |
|  | *Phoenix dactylifera* L. | [6] | GU811709 | | | | |
|  | *Typha latifolia* L. | [7] | GU195652 | | | | |
|  | *Calamus caryotoides* A. Cunn ex Mart. | [8] | JX088663 | | | | |

**References**

1. Yang JB, Tang M, Li HT, Zhang ZR, Li DZ (2013) Complete chloroplast genome of the genus *Cymbidium*: lights into the species identification, phylogenetic implications and population genetic analyses. BMC Evol Biol 13: 84.
2. Wu FH, Chan MT, Liao DC, Hsu CT, Lee YW, et al. (2010) Complete chloroplast genome of *Oncidium* Gower Ramsey and evaluation of molecular markers for identification and breeding in Oncidiinae. BMC Plant Biol 10: 68.
3. Pan IC, Liao DC, Wu FH, Daniell H, Singh ND, et al. (2012) Complete Chloroplast Genome Sequence of an Orchid Model Plant Candidate: *Erycina pusilla* Apply in Tropical Oncidium Breeding. PLoS One 7: e34738.
4. Chang CC, Lin HC, Lin IP, Chow TY, Chen HH, Chen WH, Cheng CH, Lin CY, Liu SM, Chaw SM: Chang CC, Lin HC, Lin IP, Chow TY, Chen HH, et al. (2006) The chloroplast genome of *Phalaenopsis aphrodite* (Orchidaceae): comparative analysis of evolutionary rate with that of grasses and its phylogenetic implications. Mol Biol Evol 23: 279-291.
5. Pan IC, Liao DC, Wu FH, Daniell H, Singh ND, et al. (2012) Complete Chloroplast Genome Sequence of an Orchid Model Plant Candidate: *Erycina pusilla* Apply in Tropical Oncidium Breeding. PLoS One 7: e34738.
6. Yang M, Zhang X, Liu G, Yin Y, Chen K, et al. (2010) The complete chloroplast genome sequence of date palm (*Phoenix dactylifera* L.). PLoS One 5: e12762.
7. Guisinger MM, Chumley TW, Kuehl JV, Boore JL, Jansen RK (2010) Implications of the Plastid Genome Sequence of *Typha* (Typhaceae, Poales) for Understanding Genome Evolution in Poaceae. J Mol Evol.
8. Barrett CF, Davis JI, Leebens-Mack J, Conran JG, Stevenson DW (2013) Plastid genomes and deep relationships among the commelinid monocot angiosperms. Cladistics 29: 65–87.
