# Supplementary material for: Comparative Chloroplast Genomes of Photosynthetic Orchids: Insights into Evolution of the Orchidaceae and Development of Molecular Markers for Phylogenetic Applications
Source: PLoS One. 2014 Jun 9;9(6):e99016. doi: 10.1371/journal.pone.0099016 (PMC4049609; doi:10.1371/journal.pone.0099016)
Supplement: Table S5 — RNA editing predicted in Dendrobium officinale and Cypripedium macranthos chloroplast genomes by the PREP program. (DOC) [file pone.0099016.s006.doc]

**Table S5. RNA editing predicted in *Dendrobium*** ***officinale* and** ***Cypripedium macranthos* chloroplast genomes by the PREP program.**

| Gene | Nucleotide Position | | Codon change | Editing position within codon | Amino acid change |
| --- | --- | --- | --- | --- | --- |
|  | *Cypripedium macranthos* | *Dendrobium officinale* |  |  |  |
| *acc*D | - | 329 | TCT-TTT | 2 | S-F |
|  | - | 698 | TCT-TTT | 2 | S-F |
|  | 1151 | 1139 | TCA-TTA | 2 | S-L |
|  | 1355 | - | TCA-TTA | 2 | S-L |
|  | - | 1385 | CCT-CTT | 2 | P-L |
| *atp*A | 773 | 773 | TCA-TTA | 2 | S-L^*▲☆^ |
| *atp*B | - | 1184 | TCA-TTA | 2 | S-L^▲^ |
| *atp*F | 92 | 92 | CCA-CTA | 2 | P-L^*▲☆^ |
| *atp*I | - | 428 | CCT-CTT | 2 | P-L^▲^ |
|  | - | 629 | TCA-TTA | 2 | S-L^▲^ |
| *ccs*A | - | 370 | CCC-TCC | 1 | P-S^▲^ |
|  | 647 | - | ACT-ATT | 2 | T-I |
|  | 818 | - | TCA-TTA | 2 | S-L |
| *clp*P | - | 82 | CAT-TAT | 1 | H-Y^▲^ |
|  | 559 | 559 | CAT-TAT | 2 | H-Y^*▲^ |
| *mat*K | 478 | 340 | CAT-TAT | 1 | H-Y |
|  | 943 | 781 | CAT-TAT | 1 | H-Y^*▲☆^ |
|  | - | 1129 | CAC-TAC | 1 | H-Y^▲^ |
| *ndh*B | 149 | 149 | TCA-TTA | 2 | S-L |
|  | 467 | 467 | CCA-CTA | 2 | P-L |
|  | 542 | 542 | ACG-ATG | 2 | T-M |
|  | 586 | 586 | CAT-TAT | 1 | H-Y |
|  | 611 | - | TCA-TTA | 2 | S-L |
|  | 704 | 704 | TCC-TTC | 2 | S-F |
|  | 737 | 737 | CCA-CTA | 2 | P-L |
|  | 830 | 830 | TCA-TTA | 2 | S-L |
|  | 836 | 836 | TCA-TTA | 2 | S-L |
|  | 1102 | - | CGC-TGC | 1 | R-C |
|  | 1112 | 1112 | TCA-TTA | 2 | S-L |
|  | 1193 | 1193 | TCA-TTA | 2 | S-L |
|  | 1255 | - | CAT-TAT | 1 | H-Y |
|  | 1481 | 1481 | CCA-CTA | 2 | P-L |
| *pet*B | 418 | 418 | CGG-TGG | 1 | R-W |
|  | 611 | 611 | TCA-TTA | 2 | P-L^*▲^ |
| *pet*D | - | - | - | - | - |
| *pet*G | - | - | - | - | - |
| *pet*L | - | - | - | - | - |
| *psa*B | - | 430 | CTT-TTT | 1 | L-F |
|  | - | 1256 | ACA-ATA | 2 | T-I |
| *psa*I | - | 80 | TCT-TTT | 2 | S-F |
| *psb*B | - | - | - | - | - |
| *psb*E | - | - | - | - | - |
| *psb*F | 77 | 77 | TCT-TTT | 2 | S-F^*▲^ |
| *psb*L | - | - | - | - | - |
| *rpl*2 | 2 | 2 | ACG-ATG | 2 | T-M^▲^ |
| *rpl*20 | - | 26 | ACA-ATA | 2 | T-I^▲^ |
|  | 308 | 308 | TCA-TTA | 2 | S-L^*▲^ |
| *rpl*23 | - | - | - | - | - |
| *rpo*A | 368 | 368 | TCA-TTA | 2 | S-L^*▲☆^ |
|  | - | 830 | TCA-TTA | 2 | S-L^▲^ |
| *rpo*B | 338 | 338 | TCT-TTT | 2 | S-F^*▲^ |
|  | 473 | 473 | TCG-TTG | 2 | S-L |
|  | 551 | 551 | TCA-TTA | 2 | S-L^*▲☆◇^ |
|  | 566 | 566 | TCG-TTG | 2 | S-L^*▲^ |
|  | 623 | 623 | CCG-CTG | 2 | P-L^*▲☆^ |
|  | - | 1934 | GCT-GTT | 2 | A-V^▲^ |
|  | 2000 | - | TCT-TTT | 2 | S-F |
|  | - | 2426 | TCA-TTA | 2 | S-L^▲^ |
| *rpoC*1 | 41 | 62 | CCA-CTA | 2 | P-L^*▲☆^ |
|  | 80 | - | ACC-ATC | 2 | T-I |
|  | 182 | 203 | TCT-TTT | 2 | S-F |
|  | - | 638 | TCG-TTG | 2 | S-L^▲^ |
| *rpoC*2 | 812 | - | GCC-GTC | 2 | A-V |
|  | 1081 | - | CTC-TTC | 1 | L-F |
|  | 2081 | - | ACA-ATA | 2 | T-I |
|  | 2284 | - | CGG-TGG | 1 | R-W |
|  | 3137 | - | CCA-CTA | 2 | P-L |
| *rps*2 | - | - | - | - | - |
| *rps*8 | 182 | - | TCA-TTA | 2 | S-L |
|  | 262 | - | CAT-TAT | 1 | H-Y |
| *rps*14 | 80 | - | TCA-TTA | 2 | S-L |
|  | 149 | 149 | CCA-CTA | 2 | P-L^▲^ |
| *ycf*3 | 44 | 44 | TCT-TTT | 2 | S-F^*▲☆^ |
|  | 185 | 185 | ACG-ATG | 2 | T-M |
|  | 191 | 191 | CCA-CTA | 2 | P-L^*▲☆^ |
|  | 407 | - | TCC-TTC | 2 | S-F |

‘-’: no editing;

‘^*^’: potential common RNA editing sites in genes from *Cypripedium macranthos* and Epidendroideae (*Dendrobium officinale,* *cymbidium mannii*, *Erycina pusilla*, *Phalaenopsis aphrodite*, *Phalaenopsis equestris* and *Oncidium* Gower Ramsey);

‘^▲^’: potential common RNA editing sites in genes from Epidendroideae (*cymbidium mannii*, *Dendrobium officinale*, *Erycina pusilla*, *Phalaenopsis aphrodite*, *Phalaenopsis equestris* and *Oncidium* Gower Ramsey);

‘^☆^’: potential common RNA editing sites in genes from orchids and *Cocos nucifera*;

‘^◇^’: potential common RNA editing sites in genes from orchids, *Nicotiana tabacum*, *Arabidopsis thaliana* and Poaceae.
