# Supplementary material for: Comparative Chloroplast Genomes of Photosynthetic Orchids: Insights into Evolution of the Orchidaceae and Development of Molecular Markers for Phylogenetic Applications
Source: PLoS One. 2014 Jun 9;9(6):e99016. doi: 10.1371/journal.pone.0099016 (PMC4049609; doi:10.1371/journal.pone.0099016)
Supplement: Table S6 — Sequence information for genes used in the phylogenetic analysis of the Epidendroideae. (DOC) [file pone.0099016.s007.doc]

**Table S6. Sequence information for genes used in the phylogenetic analysis of the** Epidendroideae.

| **Genes** | **Aligned length (bp)** | **Total number of sites** | **Number of polymorphic sites (*s*)** | **Parsimony information sites** | **Average number of nucleotide differences (*k*)** | **Nucleotide diversity (*Pi*)** |
| --- | --- | --- | --- | --- | --- | --- |
| *ycf*1 | 2,076 | 966 | 649 | 704 | 96.932 | 0.116 |
| *acc*D | 1,274 | 592 | 332 | 278 | 37.860 | 0.070 |
| *ccs*A | 718 | 509 | 221 | 193 | 29.599 | 0.058 |
| *mat*K | 885 | 772 | 373 | 272 | 49.415 | 0.070 |
| Combined cpDNAs | 4,593 | 2,839 | 1,575 | 1,447 | 213.806 | 0.084 |
