# Supplementary material for: Comparative Chloroplast Genomes of Photosynthetic Orchids: Insights into Evolution of the Orchidaceae and Development of Molecular Markers for Phylogenetic Applications
Source: PLoS One. 2014 Jun 9;9(6):e99016. doi: 10.1371/journal.pone.0099016 (PMC4049609; doi:10.1371/journal.pone.0099016)
Supplement: Table S7 — AT content of the ycf 1 gene in the Orchidaceae. (DOC) [file pone.0099016.s008.doc]

**Table S7. AT content of the *ycf*1 gene in** **the Orchidaceae.**

| **Taxon** | **Subfamily** | **Total length** | **AT content (%)** |
| --- | --- | --- | --- |
| *Cypripedium macranthon* | Cypripedioideae | 5,373 | 68.40 |
| *Rhizanthella gardneri* | Orchidoideae | 5,007 | 73.42 |
| *Corallorhiza striata* | Epidendroideae | 5,253 | 72.24 |
| *Cymbidium mannii* | Epidendroideae | 6,421 | 71.09 |
| *Dendrobium officinale* | Epidendroideae | 5,517 | 70.13 |
| *Erycina pusilla* | Epidendroideae | 5,316 | 71.67 |
| *Neottia nidus-avis* | Epidendroideae | 5,151 | 73.62 |
| *Oncidium* Grower Ramsey | Epidendroideae | 5,304 | 70.93 |
| *Phalaenopsis* *aphrodite* | Epidendroideae | 5,447 | 71.27 |
| *Phalaenopsis equestris* | Epidendroideae | 5,486 | 71.38 |
